# Exploring resistance to immune checkpoint inhibitors and targeted therapies in melanoma

**DOI:** 10.20517/cdr.2024.54

**Published:** 2024-10-31

**Authors:** Anum Jalil, Melissa M Donate, Jane Mattei

**Affiliations:** ^1^Department of Medicine, UT Health Science Center San Antonio, San Antonio, TA 78229, USA.; ^2^Long School of Medicine, UT Health Science Center San Antonio, San Antonio, TA 78229, USA.; ^3^Department of Hematology Oncology, UT Health Science Center San Antonio, San Antonio, TA 78229, USA.

**Keywords:** Immunotherapy, BRAF, resistance, melanoma, overcoming resistance

## Abstract

Melanoma is the most aggressive form of skin cancer, characterized by a poor prognosis, and its incidence has risen rapidly over the past 30 years. Recent therapies, notably immunotherapy and targeted therapy, have significantly improved the outcome of patients with metastatic melanoma. Previously dismal five-year survival rates of below 5% have shifted to over 50% of patients surviving the five-year mark, marking a significant shift in the landscape of melanoma treatment and survival. Unfortunately, about 50% of patients either do not respond to therapy or experience early or late relapses following an initial response. The underlying mechanisms for primary and secondary resistance to targeted therapies or immunotherapy and relapse patterns remain not fully identified. However, several molecular pathways and genetic factors have been associated with melanoma resistance to these treatments. Understanding these mechanisms paves the way for creating novel treatments that can address resistance and ultimately enhance patient outcomes in melanoma. This review explores the mechanisms behind immunotherapy and targeted therapy resistance in melanoma patients. Additionally, it describes the treatment strategies to overcome resistance, which have improved patients’ outcomes in clinical trials and practice.

## INTRODUCTION

Cutaneous melanoma, known for its rapid progression and high potential for metastasis, is strongly associated with UV exposure^[1]^. An estimated 100,640 new invasive melanoma cases will be diagnosed in 2024, increasing by 7.3%^[2]^. Until recently, metastatic melanoma was often considered a death sentence, with a five-year survival rate below 5% when treated with chemotherapy. However, in the last decade, the treatment options for melanoma have significantly advanced due to the emergence of immunotherapies and targeted therapies, including anti-CTLA4 and anti-PD-1 checkpoint inhibitors, as well as BRAF and mitogen-activated extracellular signal-regulated kinase (MEK) inhibitors^[3]^.

In March 2011, the U.S. Food and Drug Administration (FDA) approved ipilimumab, a cytotoxic T-lymphocyte associated protein 4 (CTLA-4) inhibitor, as the first immune checkpoint inhibitor (ICI) for treating metastatic melanoma^[4]^. Ipilimumab at a dose of 3 mg/kg, given either alone or in combination with a glycoprotein 100 (gp100) peptide vaccine, significantly improved overall survival (OS) in patients with previously treated unresectable stage III or IV melanoma, compared with gp100 alone, and 1- and 2-year survival rates were reported as 45.6% *vs.* 25.3% and 23.5% *vs.* 13.7%, respectively^[5]^. Since then, other immunotherapeutic agents have been approved alone or in combination. Programmed cell death 1 protein (PD-1) is another important immune regulatory pathway. Pembrolizumab (Keytruda) and nivolumab are FDA-approved inhibitors of PD-1 for advanced melanoma treatment^[6,7]^.

Combination therapy with nivolumab and ipilimumab has demonstrated superior efficacy - enhanced response rates and progression-free survival (PFS) - compared to treatment with a single checkpoint inhibitor in patients with metastatic melanoma. The double-blinded randomized controlled phase II CheckMate 069 and phase III CheckMate 067 trials showed significantly improved and durable clinical outcomes with the nivolumab plus ipilimumab combination treatment in metastatic melanoma. Notably, findings from the CheckMate 067 trial showed unprecedented results, with a median OS of 72.1 months and a 6.5-year survival rate of 56% with the combination therapy. These outcomes represent a remarkable advancement compared to the dismal median survival of just 8 months seen a decade ago^[8]^. The results of this trial led to the FDA’s approval of the combination of ipilimumab and nivolumab for the management of advanced melanoma.

The identification of the BRAF^V600E^ mutation in melanoma patients was a key development in advancing the treatment of metastatic melanoma. *BRAF* gene mutations occur in about 40%-50% of patients with advanced melanoma, leading to activation of the mitogen-activated protein kinase (MAPK) signaling pathway, increasing cellular expansion and replication^[9]^. In August 2011, the FDA approved vemurafenib as the first drug to target MAPK signaling in BRAF^V600E^-mutant melanoma. It was associated with rapid responses and short-term increased patient survival^[10]^.

MEK activity plays a pivotal role in mutant BRAF signaling, largely because MEK isoforms primarily target extracellular signal-regulated kinases (ERKs) as their catalytic substrates. This underscores the importance of MEK activity in facilitating the downstream effects of mutant BRAF, as ERKs serve as the primary conduits for transmitting these signals within the cellular environment^[11]^. Trametinib, a MEK inhibitor, was linked with improved outcomes in metastatic melanoma patients^[11]^. Several clinical trials have shown that combination treatment with first-line BRAF-MEK inhibitors in patients with BRAF V600E or V600K mutations has significantly improved outcomes. One-third of patients treated with these inhibitors achieved a 5-year survival rate, with a median OS exceeding 2 years^[12,13]^.

Despite significant progress in improving survival rates for metastatic melanoma patients through immunotherapeutic agents and targeted therapy, a notable proportion of patients still face disease progression and do not survive^[14]^. This is primarily attributed to the emergence of resistance mechanisms to these drugs. Resistance to these therapies presents a major clinical challenge, highlighting the need for deeper insight into the underlying mechanisms to develop effective new strategies to overcome it.

In this paper, we provide a comprehensive review of ICIs, examining their role in immune responses and exploring the mechanisms underlying resistance to ICIs in melanoma. Furthermore, we outline the potential events that contribute to resistance against BRAF and MEK inhibitors and propose various approaches to overcome these resistances.

## ROLE OF IMMUNE CHECKPOINTS AND THEIR INHIBITORS IN MELANOMA

### CTLA-4 and regulatory T cells

CTLA-4 is an immune checkpoint that sends a suppressive signal to T cells and regulates the activity of the immune system. Certain tumors can utilize this T cell inhibition to evade the immune response.

**Figure 1:**
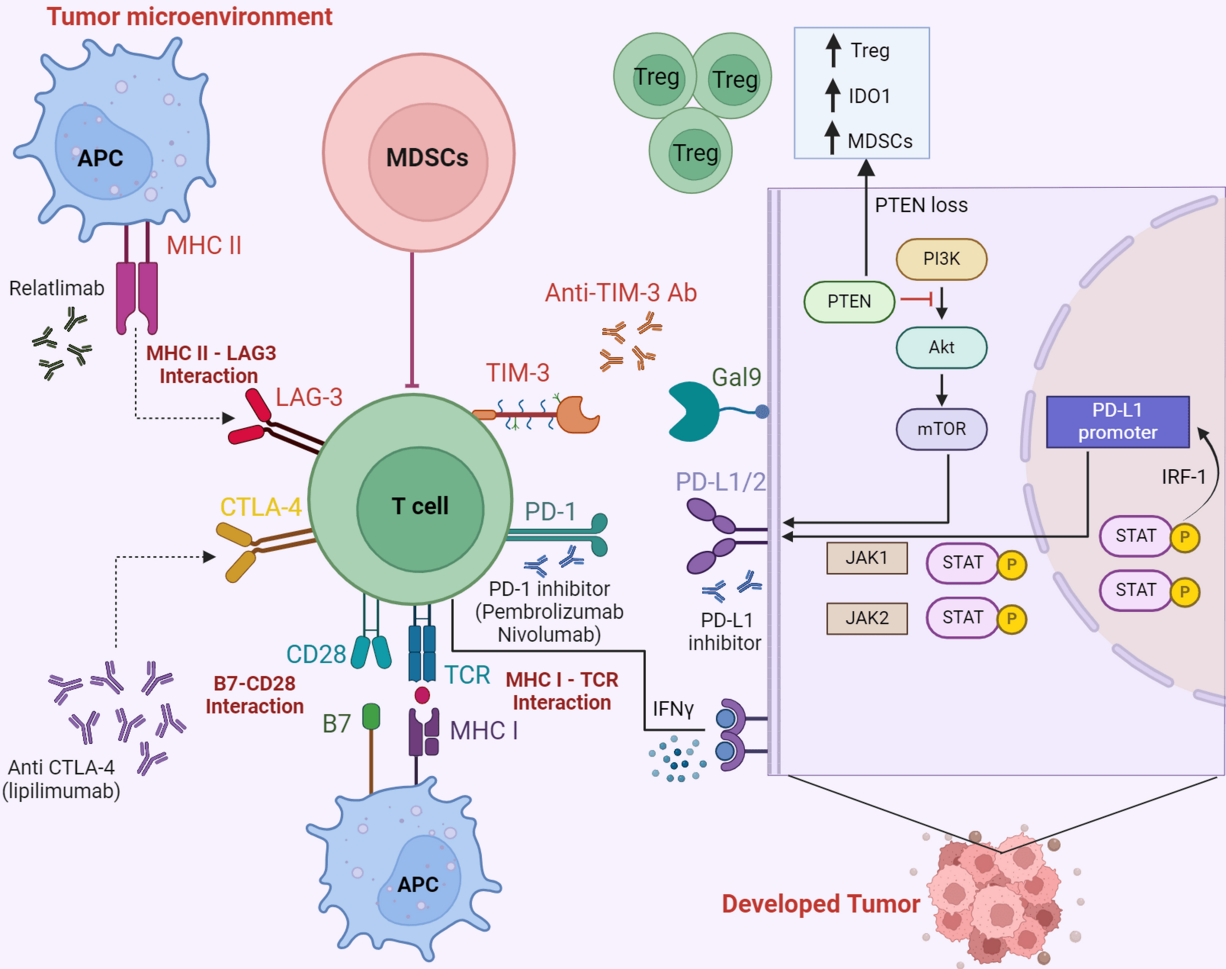
Immunotherapy and signaling pathways in melanoma. Tumor antigen processing and presentation to T cells is shown. Ipilimumab blocks CTLA-4 and B7 interaction, leading to increased immune response against tumors. PD-1 and PD-L1 inhibitors function by blocking PD-1/PD-L1 interaction, creating a cascade of immune responses that helps kill malignant cells. TIM-3 and LAG-3 are checkpoint proteins that play a role in modulating response to immunotherapy and suppressing anti-cancer immunity. Relatlimab represents a pioneering human IgG4 monoclonal blocking antibody that targets LAG-3. The PI3K-AKT-mTOR pathway is negatively regulated by PTEN in the cytoplasm. PTEN loss leads to an increase in MDSCs, T_reg_ cells, IDO1, and PD-L1 expression. IFN-γ induces PD-L1 in cancer cells through the JAK-STAT-IRF-1 pathway, as shown. LAG-3: Lymphocyte-activation gene 3; PD-1: programmed cell death protein 1; PD-L1/2: programmed cell death ligand 1 and 2; CLTA-4: cytotoxic T-lymphocyte-associated protein 4; STAT: signal transducer and activator of transcription; T_reg_: regulatory T; MDSCs: myeloid-derived suppressor cells; APC: antigen-presenting cell; TCR: T cell receptor; IRF-1: interferon regulatory factor-1; TIM-3: T cell immunoglobulin and mucin domain 3; IFN-γ: interferon-gamma; IDO: indoleamine 2,3-dioxygenase; PTEN: phosphatase and tensin homolog; PI3K: phosphatidylinositol-3-kinase; AKT: protein kinase B; mTOR: mammalian target of rapamycin; Gal9: galectin 9.

Various antigens on melanoma cells are processed by major histocompatibility complex (MHC) molecules on antigen-presenting cells (APCs) and presented to T cells, where they are detected by the T cell receptors (TCRs). However, for a complete response (CR), another co-stimulatory step is imperative: CD28 on T cells needs to interact with B7 molecules present on APCs. CTLA-4 can block CD28-B7 interaction and can instead bind to B7 molecules itself with higher affinity, thus leading to impaired T cell effector function and hindering tumor immunity. Ipilimumab is an IgG1 monoclonal antibody that blocks the interaction between CTLA-4 and B7, thereby enhancing T cell activation and boosting the immune response against tumors^[15,16]^ [Figure 1].

In addition, Regulatory T (T_reg_) cells, a subset of CD4^+^ T cells, play a crucial part in immune tolerance. T_reg_ cells can constitutively express CTLA-4, which inhibits the function of effector T cells and thereby suppresses the immune response. In addition, T_reg_ cells also suppress APCs. Ipilimumab inhibits CTLA-4, promoting T cell activation and reducing immune tolerance by T_reg_ cells.

### PD-1/PD-L1

Pembrolizumab and nivolumab target the PD-1, which is an immune checkpoint found on the surface of all activated T cells. PD-1 has an inhibitory role and regulates T cell exhaustion. This is especially important when an inflammatory response is occurring in tissues to limit autoimmunity^[17]^. When PD-1 binds to its ligands-programmed cell death ligands 1 and 2 (PD-L1 and PD-L2), it leads to the downregulation of T cells and immune tolerance. However, this role is exploited in the tumor microenvironment (TME) and is used as an immune resistance mechanism. Neoplastic cells overexpress PD-L1, which enables them to evade antitumor immunity. PD-1 and PD-L1 inhibitors function by blocking PD-1/PD-L1 interaction, which restores T cell function and creates a cascade of immune responses that helps kill malignant cells^[18-20]^.

While PD-1 blockade generally works as an immunotherapy to harness the body’s immune system against tumors, it can paradoxically accelerate cancer growth in certain cases, a phenomenon known as hyperprogressive disease (HPD). Kamada *et al.* reported that approximately 10% of advanced gastric cancer (GC) patients treated with anti-PD-1 monoclonal antibodies experienced HPD, with 4 out of 36 patients affected^[21]^. The study also showed that blocking PD-1 increased the activation of T_reg_ cells, which in turn enhanced antitumor immunity. A similar pattern has been noted in melanoma, where non-responders to anti-PD-1 treatment showed an increase in T_reg_ cells, while responders had a decrease^[22]^. In melanoma models, T_reg_ accumulation may be driven by IL-2 produced by CD8^+^ T cells, which leads to upregulation of inducible T cell costimulator (ICOS), a co-stimulatory and anti-apoptotic molecule. Additional research is required to investigate the interactions between cytotoxic T cells, T_reg_ cells, and ICOS to develop strategies that enhance the effectiveness of anti-PD-1 therapy while minimizing associated adverse events^[23]^.

## MOLECULAR MECHANISMS OF RESISTANCE TO IMMUNOTHERAPY

Mechanisms of resistance can be classified as primary or innate and acquired resistance. With primary resistance, cancer does not respond to the therapy at all, whereas relapse after an initial period of response is acquired resistance. Many intrinsic and extrinsic tumor factors lead to the development of resistance^[24]^.

While ICI enhanced survival rates in a notable number of patients with progressive melanoma, drug resistance leading to disease progression was also noted in an alarming proportion of cases. Specifically, 40%-65% of patients receiving PD-1 inhibitor therapy and over 70% of patients on anti-CTLA-4 showed minimal to zero response, likely due to primary resistance mechanisms^[25-27]^.

Several mechanisms explaining resistance to ICI have been uncovered; however, there remains a lack of comprehensive understanding for many. Tumor cells employ various mechanisms to render T cells ineffective or to escape recognition by cytotoxic T cells to evade the immune response, resulting in unchecked cellular growth and resistance to ICI. An interference by tumor cells at any of the different stages of effective immune response, including antigen presentation, T cell activation, mobilization, and infiltration of TME, can lead to tumor resistance to immunotherapy^[26]^.

### Mechanisms of primary resistance

#### PI3K/AKT pathway and upregulation of PD-L1

In many cancers, including melanoma, the expression of PD-L1 is upregulated through various pathways and is an important mediator of resistance to ICI. One such pathway involves the loss of phosphatase and tensin homolog (PTEN), which is a tumor suppressor gene. Loss of PTEN has been reported in studies as an important mechanism that increases PD-L1 expression. PTEN is responsible for the negative regulation of Phosphatidylinositol-3-kinase (PI3K)/protein kinase B (AKT)/mammalian target of rapamycin (mTOR) pathway [Figure 1].

PI3K/AKT is a crucial pathway involved in cellular proliferation and plays a role in tumorigenesis. Deletion or inactivating mutation of the *PTEN* gene leads to activation of the PI3K/AKT pathway, which increases the expression of the gene encoding PD-L1. This reduces cytotoxic T lymphocyte (CTL)-induced apoptosis of cancer cells and leads to immunoresistance^[28]^. PTEN loss and PD-L1 overexpression are linked to poorer OS, advanced tumor stage, and an increased propensity for metastasis. This mechanism has been reported in various tumor types, including melanoma, colorectal cancer (CRC), glioblastoma multiforme (GBM), and lung squamous cell carcinoma, to name a few^[29,30]^. One study reported the prevalence of PTEN alterations in melanoma cell lines at 27.6%^[31]^. Bucheit *et al.* reported that absolute loss of PTEN in stage IIIB/C melanoma patients having *BRAF* mutations is strongly associated with a notably accelerated onset of brain metastasis^[32]^.

Some other tumors, like lymphomas, upregulate PD-L1 via other pathways, including nucleophosmin/anaplastic lymphoma kinase (NPM/ALK), which activates PD-1 expression via the signal transmitter, transcription factor STAT3^[33]^. In addition, the interferons (IFNs), especially IFN-γ, also trigger the induction of PD-L1, which subsequently suppresses the activity of PD1 positive effector T cells, thus dampening the anti-cancer immune responses^[34]^.

#### Role of APC and MHC molecules and antigen presentation to T cells

Several studies have demonstrated that the increased presence of T cells or tumor-infiltrating lymphocytes (TILs) in primary melanomas predicts a more favorable prognosis, whereas the TILs correlate with a heightened likelihood of metastatic spread and grave prognosis^[35,36]^.

Tumor cells express specific tumor-associated antigens (TAA) that allow T cells to differentiate them from the host’s own cells. These antigens are recognized by T cells as foreign, thus initiating the host’s immune response to kill the invading cells. However, this process is complex and involves several critical steps that must occur for effector T cells to function properly. Any defect in these steps can lead to an ineffective immune response and confer drug resistance in tumor cells.

TCRs can identify tumor antigens only after they have been processed and presented by molecules of the MHC I present on APCs. Inside the lymph nodes, APCs present tumor antigens to T cells, leading to the generation of tumor-specific CTLs that function to kill tumor cells^[37]^.

Loss of tumor neoantigen expression can lead to a lack of T cell response. In addition, insufficient MHC expression levels can hinder antigen presentation, potentially limiting effective immune recognition within tumors and restricting the recruitment of CD4^+^ and CD8^+^ T cells^[38]^. Studies have reported loss of melanocyte antigen and loss of MHC I expression in progressive metastatic melanoma^[39]^.

In addition, previous studies have demonstrated that melanoma cells can constitutively express MHC class II molecules, and this expression is significantly upregulated in response to IFN-γ. The upregulation of MHC class II molecules on melanoma cells can lead to immune evasion via several mechanisms, such as the activation of tumor antigen-specific CD4^+^ T cells^[40]^.

#### Dendritic cell maturation impairment

Dendritic cells (DC) are an important category of APCs and hold pivotal significance in the immune response. The abundance of DCs within tumor tissues has been linked to the prognosis of patients across a spectrum of cancers. Understanding the relationship between dendritic cells (DCs) and the TME is crucial for developing immunotherapeutic strategies that aim to boost antitumor immune responses^[41]^. Impaired maturation of dendritic cells can reduce the efficacy of ICIs by limiting the upregulation of co-stimulatory molecules like CD80 and CD86, which are essential for enhancing T cell activation^[42]^.

Melanoma cells produce various factors that have immunosuppressive properties that help them escape immune response. Various cytokines can alter DC function. One such factor is interleukin-10 (IL-10), which is an immunosuppressive cytokine that can suppress the expression of antigen-presenting molecules. Gerlini *et al.* reported that IL-10 secreted by metastatic melanoma cells causes downregulation of cyclin D1 (CD1) molecules present on the surface of DCs, thus leading to ineffective antigen presentation^[43]^. The CD1 system serves as the mechanism through which T cells are presented with lipid and glycolipid antigens, which are derived from both the body’s own tissues and external sources^[44]^.

Wu *et al.* reported that IL-37b, also known as interleukin-37b, is pivotal in dampening immune responses, partly by suppressing DC maturation by downregulating the expressions of co-stimulatory molecules CD80 and CD86^[45]^.

#### Loss of function mutations in JAK 1 and 2

The Janus kinase-signal transducer and activator of transcription (JAK-STAT) pathway plays a vital role in numerous cellular functions, such as cell immunity and proliferation, and it is also involved in tumor formation. The JAK proteins are activated upon binding of cytokine ligands, which leads to post-translational modifications such as phosphorylation of cytoplasmic STAT proteins, which in turn bind DNA and regulate gene expression^[46]^.

Loss-of-function mutations in members of the JAK family of proteins, JAK1 and JAK2, have been reported as mechanisms of both primary and acquired resistance to ICIs, particularly PD-1 blockade immunotherapy, in melanoma patients. Shin *et al.* reported in 2017 that JAK1/2 loss-of-function mutations cause reduced PD-L1 expression and impaired response to IFN-γ. This genetic alteration results in primary resistance to PD-1 blockade treatment. Their research identified JAK1/2-inactivating mutations in one of 23 melanoma tumor biopsies and in two of 48 human melanoma cell lines^[47]^ [Figure 1].

#### Role of endothelins and VEGF in immunotherapy resistance

Endothelins (ET) influence tissue differentiation, repair, and growth by binding to the cell-surface ET receptors, endothelin-A (ETA) and endothelin-B (ETB). In melanocytes and malignant melanoma cells, the non-selective ETB receptor predominates among the endothelin receptors. Upon binding of ET to their receptors, various signaling pathways are activated, including protein kinase C (PKC) and MAPK pathways. Studies have shown that the ETB receptor is upregulated in melanoma cells, highlighting its potential role as a marker of melanoma progression. The upregulation of ETB receptors in melanoma cells suggests a potential role in driving melanoma progression and metastasis. Targeting the endothelin system, including ETB receptors, could be a promising therapeutic approach for melanoma treatment, either as a standalone option or alongside other treatment modalities. This might be a potential mechanism being used by tumor cells in their immune escape strategy^[26,48]^.

Vascular endothelial growth factor (VEGF) is a signaling protein released by cells that promotes the development of new blood vessels. VEGF is pivotal in promoting tumor angiogenesis, a crucial mechanism through which tumors induce the formation of new blood vessels, enabling their proliferation and potential for metastasis. Additionally, VEGF, such as VEGF-A, has been found to contribute to immune suppression within the TME. Several mechanisms have been reported in literature underlying VEGF’s contribution to immune suppression, such as its ability to suppress the function of effector T cells, augment influx of T_regs_, and their proliferation. In addition, it can cause the induction of other cells, such as myeloid-derived suppressor cells (MDSCs), leading to cancer cells escaping the immune system^[49]^. The possible involvement of VEGF-A in tumor resistance to anti-PD-1 therapy has been reported^[50]^, underscoring the fact that VEGF-A upregulation might be another mechanism employed by tumor cells in resistance to ICI therapy.

Hodi *et al.* reported immune-mediated vascular damage in biopsies analyzed from advanced melanoma cancer patients who were treated with CTLA-4 blocking antibody infusion accompanied by tumor necrosis^[51]^, highlighting the potential effect of CTLA-4 immune checkpoint inhibition on angiogenesis in the TME.

Several trials are investigating the role of Anti VEGF therapy combined with immune checkpoint blockade therapy. A Phase I study evaluating combination treatment with ipilimumab plus bevacizumab in metastatic melanoma patients was conducted in 2014 and reported a median survival of 25.1 months^[52]^. There are many other clinical trials underway to assess the efficacy of antiangiogenic therapies and immune checkpoint inhibition. One such trial is NCT01950390 (an ongoing randomized phase II trial to study the effects of ipilimumab alone or in combination with bevacizumab in patients with stage III/IV melanoma)^[53]^.

#### Indoleamine 2,3-dioxygenase expression

The cytosolic enzyme indoleamine 2,3-dioxygenase (IDO) is another factor suggested as a potential contributor to melanoma tumor immune escape mechanisms. IDO is an enzyme produced by tumor cells, as well as by dendritic cells and host macrophages, and it is responsible for catalyzing tryptophan degradation^[54]^. Insufficient tryptophan levels can suppress effector T lymphocyte proliferation. Malignant melanoma cells overexpress and upregulate IDO, which leads to cessation of T lymphocyte activity and an increase in T_regs_. This leads to tumor progression^[55]^.

Holmgaard *et al.* evaluated the combination of CTLA-4 blockade and IDO inhibition in the B16 melanoma mouse model, and data suggested that IDO inhibition can be employed to boost the effectiveness of immunotherapies in melanoma patients by augmenting T cell response. Clinical trials are currently in progress to investigate the effectiveness of anti-CTLA-4 and IDO-inhibiting therapy in melanoma patients^[56]^.

Kjeldsen *et al.* tested an immune-modulatory peptide vaccine (IO102/IO103) against indoleamine 2,3-dioxygenase (IDO) and PD ligand 1 (PD-L1) together with anti-PD-1 inhibitor nivolumab in 30 patients with metastatic melanoma in a non-randomized phase ½ study (clinical trial MM1636). Promising results were achieved, with the objective response rate (ORR) reaching 80%, 43% of patients achieving a CR, and median progression-free survival (mPFS) of 26 months, according to the report^[57]^.

### Mechanisms of acquired resistance

#### IFN-γ and JAK/STAT pathway

When T cells interact with APCs and processed tumor neoantigens, it leads to the production of IFN, especially IFN-γ. After secretion by activated T cells, IFN-γ binds to its receptor, known as IFN-γ receptor (IFNGR), which is present on cancer cells among other cell types. Upon binding, IFN-γ activates the receptor, resulting in recruitment and activation of JAK1 and JAK2, which in turn activates cytoplasmic STAT proteins. Activated STAT then forms homodimers or heterodimers with other STAT proteins and translocates into the nucleus. Inside the nucleus, these activated STAT complexes bind to specific DNA sequences in the promoters of IFN-γ-inducible genes, leading to their transcription^[58]^ [Figure 1]. This process is a key mechanism by which IFN-γ exerts its biological effects, including immune modulation and antitumor activity. Cancer cells frequently exhibit persistent activation of STAT proteins, which is linked to a poor prognosis. The STAT3 transcription factor is crucial in melanoma and many other cancer types. It contributes to oncogenesis by regulating genes that influence key cellular processes, including proliferation, inhibition of apoptosis, and metastasis^[59]^.

IFN-γ is essential in further potentiating immune response and mobilization of various cells such as macrophages and natural killer (NK) cells. On the other hand, IFN-γ also induces PD-L1 expression on melanoma cells, which binds to PD-1 on effector T cells and results in immune tolerance by suppressing the T cell function^[60-62]^. Tumor cells can utilize this mechanism to limit host immune response. Inhibiting the interaction between PD-L1 and PD-1 can revive the functional activity of T cells, boosting their ability to carry out effector functions.

Zaretsky *et al.* reported their findings in 2016 after studying biopsies of four patients with metastatic melanoma who initially responded to pembrolizumab but subsequently experienced disease progression. Data revealed loss-of-function mutations in the IFN-receptor–associated *JAK1* and *JAK2* genes. This was reported as a process underlying acquired resistance to anti-PD-1 immunotherapy in melanoma patients due to defective IFN-receptor signaling^[63]^.

#### Beta-2-microglobulin

Beta-2-microglobulin (B2M) is a subunit of MHC I molecule and its mutations have been revealed in various cancers. B2M alterations reduce the effectiveness of ICI therapy by altering antigen presentation to T cells, allowing tumors to evade immune surveillance. Previous studies have reported that the loss of functional B2M in metastatic melanoma patients leads to evasion of T cell recognition and contributes to resistance against immunotherapy^[64-66]^.

#### T cell immunoglobulin and mucin domain 3, lymphocyte activation gene 3

Several other checkpoint proteins, such as T cell immunoglobulin and mucin domain 3 (TIM-3), lymphocyte activation gene 3 (LAG-3), have been identified as crucial for modulating responses to immunotherapy. These proteins play a role in suppressing anti-cancer immunity.

TIM-3 is expressed on T helper cells, a subset of CD4^+^ T cells, and plays an inhibitory role. It interacts with its ligand, galectin 9 (Gal9), to suppress effector T cell responses by inhibiting T cell proliferation, which confers acquired resistance to ICI therapy [Figure 1]. This pathway is crucial in preventing autoimmunity; however, blocking this pathway can be a consideration for future therapeutic approaches to the treatment of cancer^[67,68]^. Koyama *et al.* reported overexpression of TIM-3 in anti-PD-1 antibody-bound CD4^+^ and CD8^+^ T cells in mouse model tumors which progressed following the initial response to PD-1 blocking treatment. Additionally, once disease progression was established in mice, they were experimentally treated with a TIM-3-blocking antibody that led to increased survival^[69]^.

LAG-3 is a molecule expressed on T cell surface. Studies have shown that LAG-3 is upregulated in PD-L1–positive melanoma, which could contribute to immunotherapy resistance^[70,71]^.

### Ways to overcome resistance to immunotherapy

Melanoma’s designation as an immunogenic malignancy is frequently linked to the detection of tumor-infiltrating lymphocytes (TILs) in excised melanoma samples^[72]^. This immunogenicity of melanoma renders it suitable for immunotherapeutic interventions. However, primary and acquired resistance to these novel therapies presents a major obstacle. Below are some approaches to combat resistance to immunotherapies.

#### Combination therapy

Using multiple therapeutic agents that target different molecular pathways is one approach to combat these resistance mechanisms. For patients with advanced melanomas harboring BRAF mutations, combination treatment with BRAF-MEK inhibitors has shown promising outcomes^[73]^. In addition, the combination of ICIs such as dual anti-CTLA-4 and anti-PD-1 therapy has been shown to have improved outcomes, especially in patients with advanced melanoma. As mentioned above, the phase III CheckMate-067 trial with nivolumab plus ipilimumab combination therapy showed remarkable clinical outcomes and improved OS rates^[8]^. Similarly, the combination of radiotherapy with anti-CTLA4 or anti-PD-L1 has been evaluated in studies, demonstrating a more effective induction of antitumor immunity against melanoma cells^[74]^.

Another therapeutic avenue being explored is combination therapy involving anti-PD-1 and LAG-3 inhibitors. In mouse cancer models, the concurrent blockade of PD-1 and LAG-3 led to tumor regression in the majority of mice with increased survival^[75]^. As mentioned above, LAG-3 is a T cell surface inhibitory molecule. It shares structural similarities with CD4 and can bind to MHC class II molecules with an affinity 100 times greater than that of CD4^[76]^. This inhibits MHC-II interaction with CD4 and TCR, resulting in suppression of T cell activation. Elevated levels of LAG-3 in T cells have the potential to shield melanoma cells and impede the apoptosis of tumor cells. LAG-3 and CTLA-4 both inhibit T cell activation and immune response against cancer^[77]^.

Relatlimab represents a pioneering human immunoglobulin G4 (IgG4) monoclonal blocking antibody that targets LAG-3 and reinvigorates the effector capabilities of depleted T cells^[78]^ [Figure 1]. The RELATIVITY-047 trial published by Tawbi *et al.* in 2022 showed that blockade of both LAG-3 and PD-1 showed significantly greater PFS than PD-1 blockade alone in untreated patients of advanced melanoma^[79]^. The trial with 714 metastatic melanoma patients showed a median PFS of 10.1 months with combination Relatlimab-nivolumab *vs.* 4.6 months for the nivolumab group (*P* = 0.006). A scoring criteria was utilized for assessing LAG-3 expression in the RELATIVITY-047 trial, and studies have shown that higher levels of LAG-3 expression demonstrate a strong connection with the response to combination immunotherapy using anti-LAG-3 and anti-PD-1^[80]^. Opdualag, a fixed-dose combination of relatlimab and nivolumab, received FDA approval on March 18, 2022, for metastatic melanoma treatment in adult and pediatric patients aged 12 and above^[81]^.

Efforts to aggressively explore further combination treatments in clinical trials are crucial for the advancement of novel and effective approaches to treating melanoma.

#### Stimulator of interferon genes protein

Stimulator of interferon genes (STING) is a protein located in the endoplasmic reticulum that triggers the release of chemokines and type I IFNs upon detecting abnormal DNA species in the cell’s cytosol. Activation of the STING pathway has been shown to enhance tumor-infiltrating lymphocyte-mediated killing of melanoma cells by increasing the expression of MHC class I molecules. However, this pathway may be impaired in various cancers, including melanomas, which reduces tumor cells’ sensitivity to T cells and can contribute to resistance to immunotherapies^[82]^.

Chipurupalli *et al.* proposed that combining a STING agonist like dimeric aminobenzimidazole (diABZI) with the BRAFi vemurafenib could effectively overcome drug resistance in melanoma treatment^[83]^.

Interestingly, while the loss of STING function in melanomas allows tumors to evade immune detection and promotes tumor growth, this same loss makes melanoma cells more susceptible to DNA viruses. This vulnerability presents an opportunity for therapeutic strategies such as oncolytic virus therapy (OVT) (e.g., T-VEC)^[84]^.

Additionally, in a B16-F10 mouse melanoma model resistant to anti-PD-1 therapy with lung metastasis, a combination of lipid nanoparticles containing STING and anti-PD-1 therapy demonstrated a synergistic antitumor effect through the induction of NK cells. Further research into STING’s role may uncover new targets for enhancing immune responses against tumors and improving treatment outcomes^[85]^.

#### Gut microbiome

Several studies have shown a significant impact of the gut microbiome on antitumor immune responses, both through innate and adaptive immunity pathways. Moreover, manipulating the gut microbiome can enhance therapeutic responses in cancer treatment. Studies with orally administered *Bifidobacterium* alone demonstrated comparable tumor control to anti-PD-L1 therapy in mouse melanoma tumor model. Moreover, the combination of both treatments almost eradicated cancer growth by effects on T cell responses and increased activation of cytotoxic T cells^[86]^.

Davar *et al.* conducted a trial in 2021 to study the effect of fecal microbiota transplantation (FMT) in combination with anti-PD-1 therapy in 16 melanoma patients who were refractory to anti-PD-1 therapy and had progressive disease^[87]^. Clinical response was seen in 6 out of 15 patients, and in these responders, there was a notable shift in the makeup of the gut microbiome toward that of the donor microbiome. *Ruminococcaceae*, *Bifidobacteriaceae*, *Lachnospiraceae*, and *Coriobacteriaceae* bacterial families were noted to be increased in the responder group after donor FMT and a decline in microbial species from the phylum *Bacteroidetes* was observed. These patients also had an increase in response to anti-PD-1 therapy, pointing toward the fact that FMT, in combination with PD-1 blockade, can be one strategy to induce antitumor immune response and overcome resistance to ICIs. Molecular analysis showed an increase in cytotoxic T cells and a decrease in circulating interleukin-8 (IL-8) in patients who had clinical responses. Elevated tumor IL-8 levels are consistently linked to adverse prognoses in various cancers, including melanoma^[88]^.

#### Histone deacetylase inhibition

Histone deacetylases (HDACs) function by eliminating acetyl groups from histone proteins, thus modulating DNA transcription and translation of genes. Irregular expression profiles of HDACs have been detected across a spectrum of malignancies^[89,90]^. In melanoma, HDACs have been shown to regulate the activity of genes related to cell growth, differentiation, and survival, among other processes. In addition, HDACs have been involved in the regulation of various genes involved in immune responses, including PD-1/PD-L1. HDAC inhibitors (HDACi) are being tested in various studies as a potential treatment option and a way to combat resistance to existing therapies^[91]^. The combination of HDACi LBH589 with anti-PD-1 therapy in mouse melanoma models resulted in increased survival rates compared to single-agent anti-PD-1^[92]^. Combining HDAC inhibitors with targeted therapies and immunotherapies could serve as effective treatments against melanoma; however, further studies are needed to evaluate efficacy and safety profile.

#### OVT

Talimogene laherparepvec (T-VEC or Imlygic^TM^) is the first FDA-approved OVT for the treatment of melanoma. T-VEC is a genetically modified herpes simplex virus type 1 (HSV-1) that has several gene deletions. These modifications enhance immunoreactivity while reducing virulence to minimize toxicity. Genetic mutations allow viruses to replicate exclusively within cancerous cells while sparing healthy ones, leading to the stimulation of both innate and adaptive systemic immune responses against tumors. In addition, the expression of granulocyte-macrophage colony-stimulating factor (GM-CSF) further augments its antitumor efficacy^[3,93]^.

Preclinical studies have demonstrated synergistic effects of oncolytic viruses (OVs) in combination with ICIs by increasing cytotoxic T cell infiltration into the TME. The use of OVT Adenovirus with anti-PD-1 antibody therapy in mouse melanoma model showed promising results, significantly reducing tumor bulk in the treated mice^[94]^.

Andtbacka *et al.* published OPTiM trial in 2015, a phase III randomized controlled trial that revealed the therapeutic benefits of OVT in melanoma for the first time and paved the way for FDA approval of T-VEC therapy for patients with advanced melanoma in 2015. The results showed that for patients with unresectable advanced melanoma, T-VEC treatment was associated with an improved durable response rate (lasting continuously for ≥ 6 months) compared to GM-CSF (16.3% *vs.* 2.1%; *P* < 0.001)^[95]^.

Later in 2022, Chesney *et al.* conducted a phase III, randomized study (MASTERKEY-265) comparing the efficacy of T-VEC combined with pembrolizumab to placebo combined with pembrolizumab in patients with advanced melanoma. However, the results showed that T-VEC combined with pembrolizumab did not significantly improve PFS or OS^[96]^.

The use of OVs is a promising antitumor strategy and its combination with ICIs can potentially be employed to overcome resistance to immunotherapies. Additional research is required to assess the effectiveness of OVs so that novel combinations with ICI can be designed.

#### Adoptive cell therapy

Adoptive cell therapy (ACT) is a form of immunotherapy that involves collecting lymphocytes (TILs) from tumor tissue, expanding this cell population, and then reinfusing them into the patient to enhance their ability to target tumor cells. The T cells are kept in an interleukin-2 (IL-2) medium, which leads to their rapid growth and proliferation^[97]^.

A multicenter phase III randomized study on 168 patients with unresectable stage IIIC-IV melanoma was conducted to compare the efficacy of ACT with TIL *vs.* ipilimumab, with the majority of patients being refractory to anti-PD1 therapy. Promising results showed a median PFS of 7.2 *vs.* 3.1 months with TIL therapy *vs.* ipilimumab alone. However, grade ≥ 3 adverse events occurred in all TIL patients compared to 57% of patients who were given ipilimumab alone^[98]^. ACT has encouraging potential and could offer a viable treatment alternative for patients who are unresponsive or resistant to current ICI treatments.

### Molecular mechanisms of resistance to targeted therapies

A mutation in the *BRAF* gene is present in about 40%-60% of melanoma cases, with 80%-90% being a missense V600E mutation, leading to a conformational change in the BRAF protein. This change mimics the phosphorylation of its activation site, resulting in increased rat sarcoma protein (RAS)-independent kinase activity and subsequent activation of the MAPK/ERK pathway^[99,100]^. Although it normally requires dimerization of RAS for the initiation of the MAPK pathway, this mutation functions as an activated monomer, bypassing this requirement for signal transduction^[101]^. Therapies targeting mutant BRAF, such as the BRAF inhibitors (BRAFi) vemurafenib, dabrafenib, and encorafenib and the MEK inhibitors (MEKi) trametinib and cobimetinib, result in kinase inhibition and subsequent inactivation of the MAPK pathway, causing cell cycle arrest and increased apoptosis^[11,102]^.

Significant improvements in patient outcomes have been recognized with the usage of combination treatment with BRAF inhibitors and MEK inhibitors. However, resistance to these therapies has been noted in patients who either do not respond to treatment at all, referred to as primary resistance, or show initial improvement followed by eventual recurrence, known as acquired resistance. Primary resistance to BRAFis and MEKis has been observed in about 15%-20% of patients, with a multiplicity of proposed mechanisms that are also associated with acquired resistance to treatment, such as tumor heterogeneity and loss of tumor suppressor genes^[100,103]^. Notably, with the use of a single BRAFi agent, the average PFS, or acquired resistance, upon therapy initiation is 6-8 months^[104]^. Combination therapies with a MEKi, i.e., dabrafenib and trametinib, improved PFS to 12 months^[105]^. In the COLUMBUS study, new-generation BRAFis and MEKis, such as encorafenib and binimetinib, respectively, have shown a superior mPFS of 14.9 months (95%CI 11.0-18.5) when used in combination, compared to the older-generation agent vemurafenib, which had an mPFS of 7.3 months (95%CI 0.41-0.71)^[106]^. The majority, up to 80%, of resistance cases are due to the reactivation of the MAPK pathway, with a lesser extent involving the activation of alternative pathways^[100,107]^.

#### Tumor heterogeneity and plasticity

Tumor heterogeneity can arise inherently, through the acquisition of genetic mutations, or via selection pressure while receiving treatment. Increased levels of AXL receptor tyrosine kinase (AXL), epidermal growth factor receptor (EGFR), and Wnt family member 5A (WNT5A) have been shown to confer primary treatment resistance, with the upregulation of these genes following initiation due to selective pressure of certain mutations induced by the treatment itself^[107,108]^.

Growth factors secreted by stromal cells, like fibroblasts and macrophages, encourage innate resistance to BRAFi. Increased collagen in the extracellular membrane (ECM) favors tumor cell proliferation and progression due to the increasing rigidity of the ECM. Transforming growth factor-β (TGF-β) secreted from fibroblasts regulates the stiffness of collagen, consequently regulating nuclear yes-associated protein (YAP) localization and cell adhesion. YAP/TAZ (transcriptional coactivator with PDZ-binding motif) activation has been linked with tumor progression, with strong associations of the de-differentiated and invasive melanoma phenotypes^[107]^.

**Figure 2:**
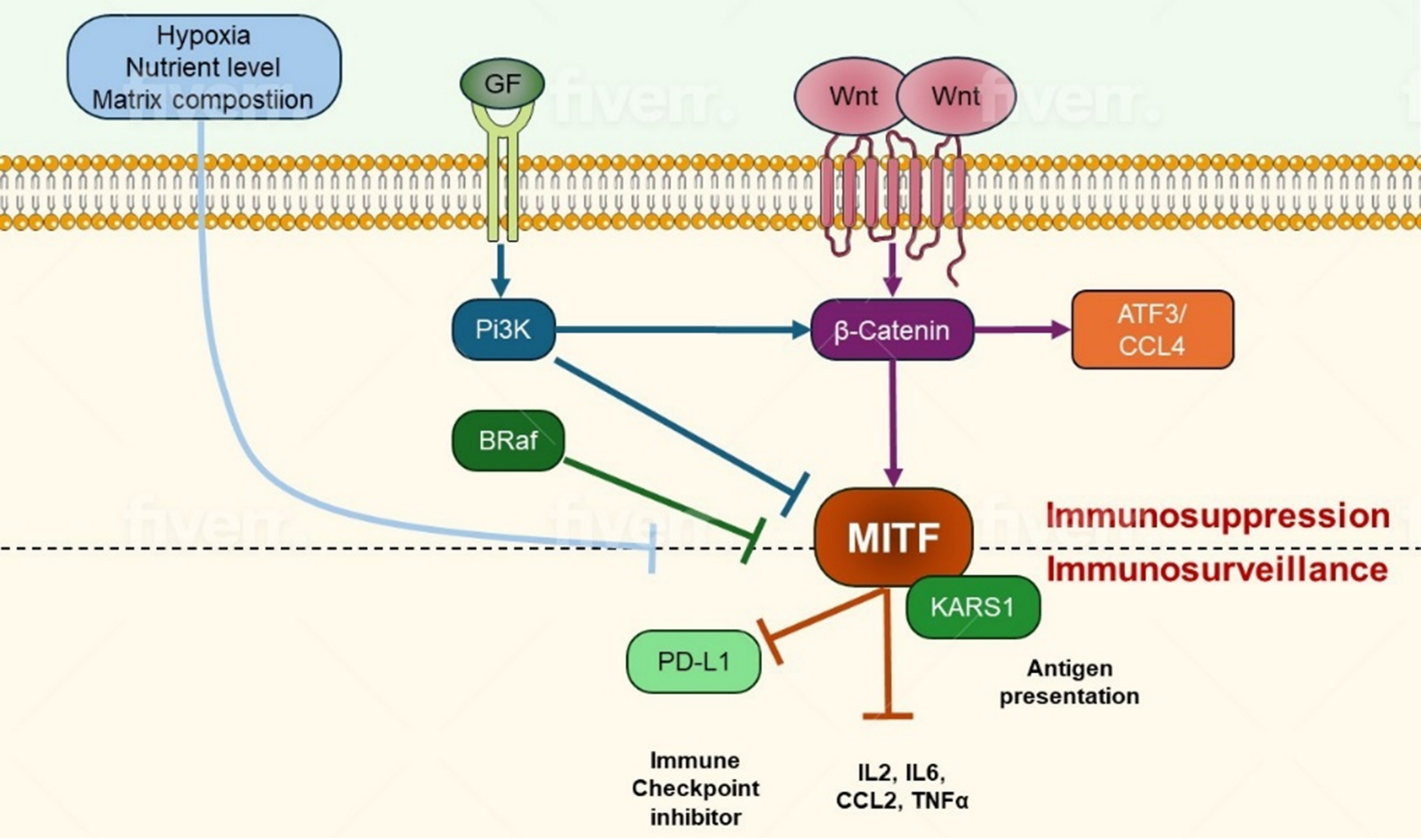
Signaling pathways regulating the activity and levels of MITF, a key transcription factor. MITF: Microphthalmia-associated transcription factor; PD-L1: programmed death-ligand 1; PI3K: phosphoinositide 3-kinase; ATF3/CCL4: activating transcription factor 3/macrophage inflammatory protein-1 beta; Wnt: signaling proteins; GF: growth factor receptor.

Phenotype switching occurs as a result of the TME to escape inhibition by targeted therapies. Tumor heterogeneity associated with microphthalmia-associated transcription factor (MITF) is common, with varied levels conferring different behaviors to melanoma tumors and cells^[62,100]^. High and low levels of MITF have been found to reduce the effect of BRAFi, alluding to the idea of phenotype switching depending on the TME. MITF levels demonstrate an inverse correlation with AXL expression, often characterized by the common phenotype MITFlow/AXLhigh. This phenotype has been implicated in acquired resistance, as tumor cells transition from a proliferative state to an invasive one, thereby facilitating metastasis^[62,103]^ [Figure 2].

**Figure 3:**
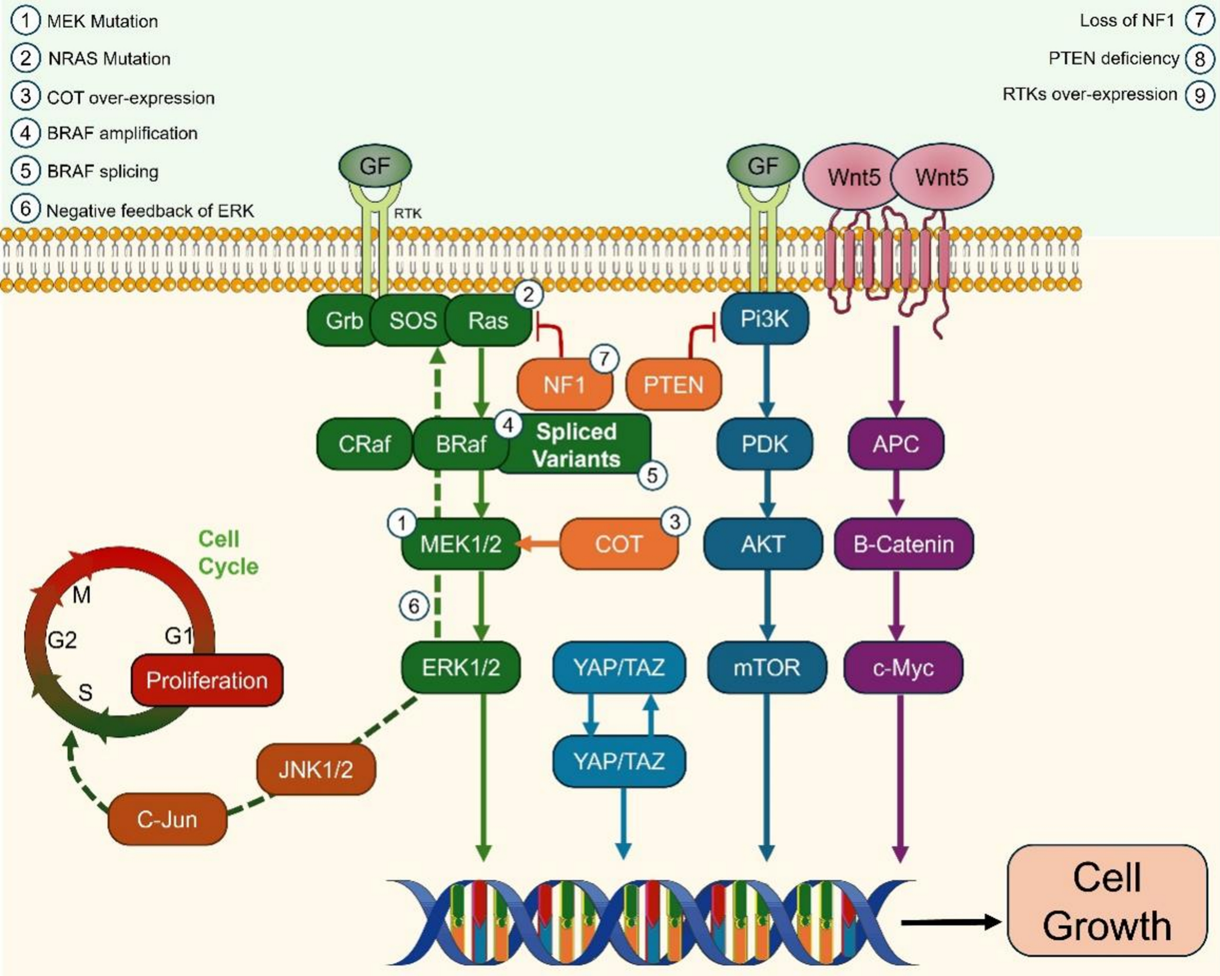
Schematic summary of mechanisms underlying resistance to target therapy. PI3K: Phosphoinositide 3-kinase; PDK: phosphoinositide-dependent kinase; AKT(PKB): protein kinase B; mTOR: mammalian target of rapamycin; APC: adenomatous polyposis coli; ERK: extracellular signal-regulated kinase; MEK: mitogen-activated extracellular signal-regulated kinase; NK1/2: c-Jun N-terminal kinases; cRaf: family of serine/threonine kinases; Grb: growth factor receptor-bound protein; GF: growth factors; YAP/TAZ: yes-associated protein and transcriptional coactivator with PDZ-binding motif.

The c-JUN (Jun-Proto-Oncogene, AP1) / JNK (c-Jun N-terminal kinases, stress-activated protein kinases) pathway plays a role in the regulation of apoptosis through cellular stress responses, as well as in other key cellular processes such as differentiation and inflammation^[109]^. Similarly to the mechanism described above, the TME is modulated by the interactions between MITF and the c-JUN protein (MITFlow/c-JUNhigh) to produce inflammatory cytokines, inducing melanoma de-differentiation, and subsequently recruiting myeloid immune-suppressive cells into the TME to facilitate tumor progression, resistance to targeted therapies, and metastasis^[110,111]^ [Figure 3].

#### microRNA-mediated resistance mechanisms

The aberrant expression of microRNAs (miRNAs) also plays a role in developing drug resistance to BRAFis and MEKis through a multitude of mechanisms due to the involvement of transcriptional regulation in multiple genes. These mechanisms include alternative signaling pathways, TME modulation, cell proliferation and survival, and apoptosis^[112]^.

The expression of miRNAs miR-204-5p and miR-211-5p has been observed to directly influence the regulation of MAPK and alternative pathways in cell proliferation and survival. When introduced to short-term BRAFi treatment, these miRNAs are upregulated to induce reactivation of MAPK or PI3K/AKT/mTOR pathways by induction of transcription factors signal transducer and activator of transcription 3 (STAT3) and MITF, respectively^[113,114]^ [Figure 2].

Response to BRAFis is also impacted by the influence of miRNAs on the TME, including factors such as angiogenesis, inflammation, and immune evasion^[103]^. Vergani *et al.* reported an association between deregulated miRNAs miR-34a, miR-100, and miR-125b and increased monocyte chemoattractant protein-1 (CCL2) production in short-term BRAFi treatment. The increased production of CCL2, a potent chemokine expressed in the TME, was found to be directly involved in the proliferation of melanoma cells and resistance to apoptosis via NF-κB signaling and hypoxia-inducible factor 1 (HIF1) transcriptional regulation^[115]^.

Tumor progression has been associated with the pro-inflammatory mediator cyclooxygenase-2 (COX2), which is regulated by the miRNA miR-146a-5p (miR-146a). miR-146a negatively regulates the nuclear factor kappa-light-chain-enhancer of activated B (NF-κB) signaling pathway via downregulation of tumor necrosis factor receptor-associated factor 6 (TRAF6), thereby blocking the activation of factors that contribute to the survival of melanoma cells. One study reported an induced decrease in miR-146a expression and increased expression of COX2 with chronic exposure to BRAFis, enabling melanoma cell growth and survival through acquired resistance^[116]^.

#### MAPK reactivation

Alterations in genes such as BRAF, neuroblastoma RAS viral oncogene homolog (NRAS), MEK1/MEK2, and neurofibromin-1 (NF1) can induce MAPK reactivation, resulting in sustained ERK signaling to promote cellular proliferation^[100,117]^. These alterations confer primary and acquired resistance, emphasizing the importance of the MAPK pathway for cellular survival. BRAFi-induced reactivation of the MAPK pathway has been observed where the MAPK pathway is blocked in mutant cells but activated in non-mutant cells through rapidly accelerated fibrosarcoma (RAF) dimerization of the drug-free RAF protein. The mechanisms underlying this phenomenon include BRAF amplification, alternative BRAF splicing, and overexpression of different RAF isoforms, like RAF proto-oncogene serine/threonine-protein kinase (CRAF). The overexpression of certain RAF isoforms, like CRAF, occurs when BRAF is inhibited to maintain the activity of the MAPK pathway. The loss of the negative regulator of RAS, the NF1 tumor suppressor gene, also activates CRAF to further propagate signal transduction via the MAPK pathway^[103]^ [Figure 3].

MEK can be directly activated by CRAF expression, as well as in a RAF-independent manner via protein kinase cancer Osaka thyroid (COT, also known as TPL2, MAP3K8) to activate the MAPK pathway. Elevated COT levels are frequently seen in BRAFi-resistant tumors and are responsible for about 10% of de-novo mutations in resistant mutant melanoma cases^[118]^. Increased levels of COT have been associated with depleting levels of BRAF V600E in the setting of BRAFi, highlighting the possible antagonization of COT expression by BRAF and its ability to reactivate MAPK signaling^[103,117,119]^.

The PI3K/AKT/mTOR pathway and the MAPK/ERK pathway are interrelated; they cross-communicate, allowing for signal transduction to continue through the upregulation of one pathway when the other is blocked^[100]^. As mentioned above, this pathway also plays a role in both immunotherapy and targeted therapy resistance through a similar mechanism. Other mechanisms that increase PI3K and subsequent MAPK signaling include hepatocyte growth factor (HGF) release by stromal cells, activating mesenchymal-epithelial transition factor (MET); PI3K/AKT/mTOR activating mutations to increase anti-apoptotic signals; increased levels of receptor tyrosine kinases (RTK) by decreased proteolytic shedding or upregulation of growth factors that bind RTKs, resulting in activation and persistent signaling^[62,100]^ [Figure 3].

#### Alternative splicing and amplification of BRAF

Alternative splicing of BRAF^V600E^ produces splicing variants, some of which lack the RAS binding domain (RBD) due to the absence of select exons and subsequently form dimers regardless of RAS activation. These variants can form homodimers (BRAF-BRAF) and heterodimers (BRAF-CRAF) that do not require activation via upstream signals due to their retained signaling capacity. Up to 30% of BRAFi treatment-resistant cases are due to these variants, as BRAFis inhibit monomeric BRAF, although still susceptible to MEKi^[107]^. Previous studies have identified multiple splice variants that exhibit resistance to vemurafenib and dabrafenib, such as p61 BRAF V600E splicing variant protein, which lacks exons 4-8^[120,121]^.

The amplification of the mutant BRAF^V600E^ allele can lead to its overexpression, rendering BRAFi ineffective in suppressing elevated BRAF levels, and thus conferring drug-saturable acquired resistance, where administering increasing amounts of BRAFi can overcome treatment failure. This increased level also causes spontaneous dimerization of the mutant BRAF protein, thus re-activating MAPK/ERK signaling^[117]^.

Resistance due to dimerization of RAF has prompted the development pan-RAF inhibitors, targeting all three RAF isoforms, such as BRAF, CRAF, and ARAF. Preclinical trials of pan-RAF inhibitors, such as LY3009120, have been proposed to combat resistance in such cases, although clinical efficacy was limited when used as monotherapy^[122,123]^. Several clinical trials are currently investigating combination therapies with pan-RAF inhibitors and other targeted therapies, such as MEKis (NCT03905148, NCT04985604, NCT04835805). A randomized multi-arm phase II clinical study evaluating the pan-RAF inhibitor naporafenib in combination with either an ERK inhibitor, a MEKi, or a CDK 4/6 inhibitor is also currently being investigated (NCT04417621)^[124]^ [Figure 3].

### Ways to overcome resistance to targeted therapies

The reactivation of the MAPK pathway is the predominant mechanism through which both primary and acquired resistance to targeted therapies develops. First-line treatment, according to the National Comprehensive Cancer Network (NCCN) melanoma guidelines, recommends a combination therapy of BRAFi and MEKi for those with the activating BRAF V600 mutation and metastatic cutaneous melanoma^[125]^.

Potential strategies to overcome resistance include blocking the MAPK pathway further downstream at ERK, suggesting the usage of ERK inhibitors to effectively block this pathway^[126]^. Targeting the alternate pathway PI3K/AKT/mTOR to effectively inhibit signal propagation has also been elucidated, with clinical trials evaluating the use of PI3K inhibitors in conjunction with BRAFi and MEKi combination therapy. Alternative targets in this pathway were necessitated due to the significant toxicities and rapid clearance of PI3K inhibitors; thus, preclinical trials are currently investigating the use of mTOR inhibitors in combination with BRAFi to induce apoptosis^[127,128]^.

Other mechanisms targeting apoptosis include targeting molecules cyclin-dependent kinase (CDK) 4/6, HDAC, heat shock protein 90 (HSP90), and B-cell lymphoma 2 (BCL-2) family protein in combination with BRAFi/MEKi^[108]^. These listed mechanisms are currently undergoing clinical trials to investigate their efficacy in each respective proposed mechanism. Louveau *et al.* reported clinical benefits in melanoma patients with cyclin-dependent kinase inhibitor 2A (CDKN2A) loss when treated with a combination of CDK 4/6 inhibitors and BRAFis^[129]^. The ongoing clinical trial NCT02645149 is investigating matched therapies based on tumor genetic profile, including the utilization of combined BRAFi/MEKi alongside CDK 4/6 inhibitor therapy in patients with CDKN2A deletions.

As noted earlier, HDAC inhibitors have been investigated in several phase I clinical trials (NCT02836548, NCT00667082) either as monotherapy, in combination with a BRAFi and/or MEKi, or in combination with other agents like proteasome inhibitors^[94,95,130]^. HSP90 involvement in acquired resistance has been demonstrated by Eroglu *et al.*, with preclinical trials indicating the potential to reverse BRAFi and BRAFi/MEKi resistance^[131]^.

Clinical trials exploring a triad therapy of BRAFi, MEKi, and HSP90 inhibitors, such as XL888 (NCT02721459) and AT13387 (NCT02097225), are currently underway. NCT02836548 is a phase I/II ongoing clinical trial evaluating the efficacy and side effects of BCL-2 inhibitor Navitoclax in conjunction with dabrafenib and trametinib to target the apoptosis regulator BCL-2, which is typically overexpressed in melanoma^[132]^.

Persistent signaling due to the upregulation of RTK is another area of potential study, specifically targeting RTK with inhibitors in combination with BRAFI/MEKi. Invasive melanoma phenotype has been associated with the signaling of steroid receptor coactivator (SRC), focal adhesion kinase (FAK), and STAT3, which are activated by the upregulation of RTK, posing other sites of potential inhibitors in conjunction with MAPKi for this specific phenotype^[133]^. Alternative therapies targeting AXL and paired box gene 3 (*PAX3*)-mediated MITF upregulation to overcome resistance have also been suggested^[134,135]^.

p21-Activated kinase 1 (PAK1) inhibition is another pathway that should be further explored as a potential target to enhance sensitivity to targeted therapies in melanoma^[136]^. PAKs are a group of serine/threonine protein kinases that are crucial for signal transduction and cytoskeletal dynamics. PAKs are effector proteins of Rho GTPases, particularly Rac and Cdc42^[137]^. Studies have shown that activation of PAK1 can impair the detection of DNA damage in melanocytes. In healthy cells, this pathway helps melanocytes tolerate DNA damage caused by UV exposure. However, in melanoma, PAK1 activation contributes to tumor cells’ resistance to DNA-damaging agents, enhancing their tolerance to damage and promoting resistance to conventional chemotherapeutic agents^[138]^. Additionally, inhibiting PAK1 could enhance the efficacy of targeted therapies as well. PAK1 has been found to interact with the PI3K/AKT pathway as well as the MAPK/ERK pathway. Increased activation of PAK1 in cancer cells has been implicated in conferring BRAFi resistance^[139,140]^.

### Combination of ICIs and targeted therapies for melanoma treatment

There is growing interest in exploring combination therapy using targeted therapies and ICIs to address resistance to targeted and immunotherapies in melanoma. This approach could offer a promising avenue for improving treatment efficacy and addressing resistance issues.

Preclinical studies have shown that combining BRAF/MEK inhibitors with ICIs leads to better responses and outcomes. In mouse melanoma models with BRAF^V600E^, the MEK inhibitor trametinib demonstrated enhanced antitumor activity when used alongside immunotherapy and the BRAF inhibitor dabrafenib^[141]^. Previous research has also indicated that BRAF inhibition boosts the expression of melanocyte differentiation antigens (MDA) and MHC class I, which improves the detection of tumor cells by cytotoxic T cells^[142,143]^. While combination therapy shows great promise, it is essential to carefully consider the potential toxicity of these drugs, as it could be a limiting factor.

Initial trials combining ipilimumab with vemurafenib reported hepatotoxicity and were terminated^[144]^. Similarly, the combination of ipilimumab with dabrafenib and trametinib was linked to significant gastrointestinal adverse effects^[145,146]^.

A Phase Ib study (NCT01656642) by Sullivan *et al.* investigated the combination of atezolizumab (anti-PD-L1) with either vemurafenib alone or vemurafenib plus cobimetinib in patients with BRAFV600-mutated metastatic melanoma^[147]^. The triple therapy achieved an objective response rate of 71.8% and showed an increased median duration of response (DOR) of 17.4 months compared to 10.6 months with the other regimen. Biomarker data also indicated that the run-in phase with cobimetinib plus vemurafenib was linked with a rise in activated CD4^+^ T-helper cells. Subsequently, a phase III study, IMspire150 (NCT02908672), was done to further evaluate the combination of atezolizumab, vemurafenib, and cobimetinib. Results showed a tolerable safety profile with triple therapy and increased PFS in patients with BRAF^V600^ advanced melanoma^[148]^.

The KEYNOTE-022 trial (NCT02130466) was a five-part randomized phase I/II trial that compared the combination of pembrolizumab with dabrafenib and trametinib (triplet therapy) to dabrafenib and trametinib alone (doublet therapy). At a median follow-up time of 9.6 months, the triplet arm showed numerically better PFS compared to the doublet arm (16.0 *vs.* 10.3 months; HR 0.66; *P* = 0.043), though the primary endpoint did not reach statistical significance^[149]^. A follow-up analysis at 36.6 months revealed significant improvements in PFS, OS, and DOR. Median PFS increased to 16.9 months compared to 10.7 months (HR 0.53; 95%CI 0.34 to 0.83)^[150]^.

COMBI-i (NCT02967692)^[151]^ is a three-part randomized phase III trial testing investigational anti-PD-1 antibody spartalizumab in combination with dabrafenib plus trametinib (Sparta-DabTram) *vs.* placebo plus dabrafenib and trametinib (placebo-DabTram) in patients with *BRAF* V600 advanced melanoma. The objective response rate in parts 1 (safety run-in) and 2 (biomarker cohort) of COMBI-i, with a median follow-up of 24.3 months and including 36 patients, was 78%, with 44% achieving a CR. However, in COMBI-i part 3, the primary endpoint of improved investigator-assessed PFS was not met. Additionally, patients receiving Sparta-DabTram experienced more treatment-related adverse effects compared to those receiving the placebo.

Several other clinical trials are actively investigating the most effective strategies for sequencing and combining targeted therapies with ICIs in melanoma. There is a significant need to explore additional combination options, optimize treatment sequencing, and fine-tune dosing regimens, especially in light of resistance challenges.

## CONCLUSION

Over the past decade, substantial advancements in immunotherapy and targeted therapy have significantly improved survival outcomes for melanoma patients. However, approximately 50% of these patients still experience disease progression and ultimately succumb to the disease, with a considerable proportion of cases attributed to resistance against current therapies. Recent progress in both basic and clinical research has deepened our insight into the molecular mechanisms driving resistance to immunotherapy and targeted therapy in metastatic melanoma.

Studies have identified critical pathways and genetic alterations that contribute to therapeutic resistance, shedding light on the complex TME and the immune evasion strategies employed by melanoma cells. This review highlights several key pathways involved in resistance in advanced melanoma, including the PI3K/AKT/mTOR and JAK-STAT pathways, as well as the crucial roles of APCs, MHC molecules, IFN-γ, VEGF, and endothelins, among others.

While combination therapies, such as immunotherapy paired with targeted therapy, can initially show effectiveness by addressing various mechanisms driving cancer growth, resistance often develops as the cancer adapts and evolves. Future investigations should focus on integrating diverse approaches, identifying novel biomarkers for patient stratification, and developing drug combinations that simultaneously target multiple resistance mechanisms.

## DECLARATIONS

### Copyright

The author(s) 2024.
